# (ADP-ribosyl)hydrolases: structure, function, and biology

**DOI:** 10.1101/gad.334631.119

**Published:** 2020-03-01

**Authors:** Johannes Gregor Matthias Rack, Luca Palazzo, Ivan Ahel

**Affiliations:** 1 Sir William Dunn School of Pathology, University of Oxford, Oxford OX1 3RE, United Kingdom;; 2 Institute for the Experimental Endocrinology and Oncology, National Research Council of Italy, 80145 Naples, Italy

**Keywords:** macrodomain, ARH3, DraG, catalytic mechanism, structural biology, genome stability, ADP-ribose, ADP-ribosylation, DNA damage, PARG, PARP

## Abstract

ADP-ribosylation is an intricate and versatile posttranslational modification involved in the regulation of a vast variety of cellular processes in all kingdoms of life. Its complexity derives from the varied range of different chemical linkages, including to several amino acid side chains as well as nucleic acids termini and bases, it can adopt. In this review, we provide an overview of the different families of (ADP-ribosyl)hydrolases. We discuss their molecular functions, physiological roles, and influence on human health and disease. Together, the accumulated data support the increasingly compelling view that (ADP-ribosyl)hydrolases are a vital element within ADP-ribosyl signaling pathways and they hold the potential for novel therapeutic approaches as well as a deeper understanding of ADP-ribosylation as a whole.

## Abstract (precis)

In this review, Rack et al. provide an overview of the different families of (ADP- ribosyl)hydrolases and discuss their molecular functions, physiological roles, and influence on human health and disease.

Posttranslational modifications (PTMs) of proteins provide efficient ways to fine-tune or repurpose protein functions by altering their activities, localization, stability, or interaction networks. PTMs thus allow organisms to adapt rapidly to changes in their environment, including nutrient availability or exposure to chemotoxins, or transition between environments, as in the case of a microbial pathogen entering a host body. Consequently, the function of PTMs can be conceived as expanding the limited genome-encoded proteome—typically only a few thousand proteins—to millions of distinct protein forms.

## ADP-ribosylation—intricate and versatile

**Figure 1.**
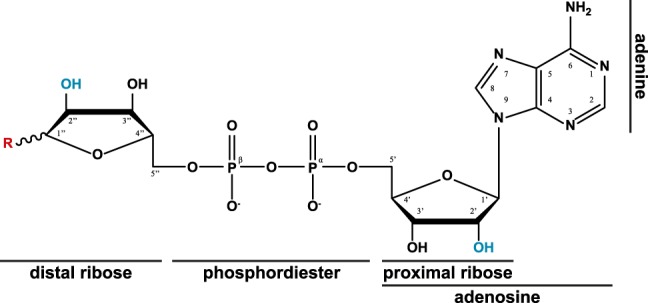
The chemical structure of ADP ribose, including atom and substructure labels, as used in this review. Throughout the ADP-ribosylation cycle different moieties (R, red) are attached to the anomeric carbon (C1′′); namely, the substrate β-NAD^+^ (nicotinamide is linked *trans* relative to the 2′′OH), the formed reaction products (linked *cis* [α] relative to the 2′′OH) (Table 1), and ADPr (1′′OH; anomeric mixture in aqueous solution). Linkage sites of consecutive ADP-ribose moieties within PAR are highlighted in blue ([2′] linear linkage; [2′′] branch point linkage).

ADP-ribosylation is an ancient PTM and intrinsically links signaling with basic metabolism. The modification is established by the transfer of a single or multiple ADP-ribose (ADPr) unit(s) from the redox cofactor β-nicotinamide adenine dinucleotide (β-NAD^+^) onto a variety of acceptor residues on the target protein (Table 1; Fig. 1). Diversification of NAD^+^ signaling is particularly apparent in vertebrata, being linked to evolutionary optimization of NAD^+^ biosynthesis and increased (ADP-ribosyl) signaling (Bockwoldt et al. 2019). ADP-ribosylation is used by organisms from all kingdoms of life and some viruses (Perina et al. 2014; Aravind et al. 2015) and controls a wide range of cellular processes such as DNA repair, transcription, cell division, protein degradation, and stress response to name a few (Bock and Chang 2016; Gupte et al. 2017; Palazzo et al. 2017; Rechkunova et al. 2019). In addition to proteins, several in vitro observations strongly suggest that nucleic acids, both DNA and RNA, can be targets of ADP-ribosylation (Nakano et al. 2015; Jankevicius et al. 2016; Talhaoui et al. 2016; Munnur and Ahel 2017; Munnur et al. 2019).

**Table 1.**
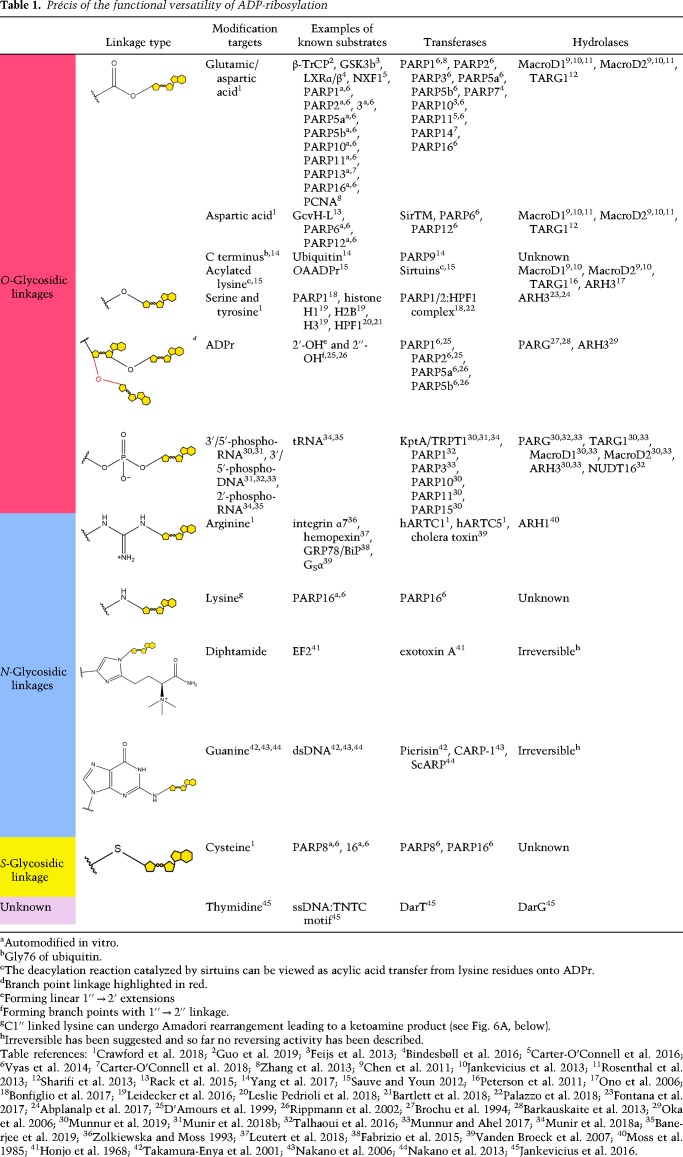
Précis of the functional versatility of ADP-ribosylation

The ADP-ribosylation reaction is catalyzed by a diverse range of (ADP-ribosyl)transferases (ARTs). Phylogenetically, their catalytic domains are part of the ADP-ribosyl superfamily (Pfam clan CL0084) (Amé et al. 2004) and three main clades are generally distinguished based on their characteristic catalytic motif: (1) the H-H-Φ clade, containing TRPT1/KtpA (also termed Tpt1); (2) the R-S-E clade, containing the cholera toxin-like ARTs (ARTCs); and (3) the H-Y-[EDQ] clade, including the diphtheria toxin-like ARTs (ARTDs) (Aravind et al. 2015). [Sequence motifs are given following the regular expression syntax of the ELM resource (http://www.elm.eu.org; Aasland et al. 2002; Gouw et al. 2018).] Functionally, the majority of ARTs catalyze the transfer of a single ADPr moiety onto an acceptor site, termed mono(ADP-ribosyl)ation (MARylation). For example, ARTCs are mostly arginine-specific mono(ADP-ribosyl)transferases with the exception of a small group of guanine-specific ADP-ribosylating toxins found in some cabbage butterfly and shellfish species (Table 1; Takamura-Enya et al. 2001; Nakano et al. 2015; Crawford et al. 2018). ARTDs (including the best characterized class poly(ADP-ribosyl)polymerases [PARPs]) appear to have a comparatively broad target range with acidic (glutamate/aspartate), thiol (cysteine), and hydroxyl (serine/tyrosine)-containing residues among others being described as acceptors (Table 1). Lastly, TRPT1/KptA and several mammalian PARPs have been found to modify the termini of phosphorylated nucleic acids (Talhaoui et al. 2016; Munnur and Ahel 2017; Munir et al. 2018b; Munnur et al. 2019).

In addition to these intrinsic specificities, recent studies have highlighted that the target preference of some transferases can be altered depending on the cellular context. For example, PARP1 and 2 (PARP1/2) catalyze primarily the modification of acidic residues via ester-type *O*-glycosidic linkages in vitro. However, the main type of ADP-ribosylation produced by PARP1/2 in response to DNA damage is the modification of serine residues through an ether-type *O*-glycosidic linkage (Table 1; Leidecker et al. 2016; Fontana et al. 2017; Larsen et al. 2018; Palazzo et al. 2018). This discrepancy was reconciled by the discovery of the auxiliary histone PARylation factor 1 (HPF1), which interacts with PARP1/2 and induces the observed switch in activity (Gibbs-Seymour et al. 2016; Bonfiglio et al. 2017; Palazzo et al. 2018). Further evidence suggests that the PARP1/2:HPF1 interaction may also enable synthesis of tyrosine-linked ADP-ribosylation (Bartlett et al. 2018; Leslie Pedrioli et al. 2018).

Apart from mono(ADP-ribosyl)ation (MARylation), PARP1, PARP2, and PARP5a/b (tankyrase-1/2) were shown to synthesize linear ADP-ribose polymers, termed poly(ADP-ribosyl)ation (PARylation), with a ribose(1′′ → 2′)ribose-phosphate-phosphate backbone (Fig. 1; Table 1; D'Amours et al. 1999; Vyas et al. 2014). In addition, PARP1/2 can infrequently (<3 mol%) introduce branch points with ribose(1′′ → 2′)ribose(1′′ → 2′′)ribose structures into the polymer (Alvarez-Gonzalez and Jacobson 1987; D'Amours et al. 1999; Chen et al. 2018). Together, this spectrum of different modification sites and types makes ADP-ribosylation one of the most intricate and versatile PTMs.

### Beyond PTMs: ADP-ribosylation of nucleic acids

Despite their evolutionary separation, TRPT1/KptA-type transferases are sometimes classified as the 18th PARP. KptA was first characterized in yeast as a tRNA 2′-phosphotransferase that removes the 2′-phosphate at the splice junction generated by fungal tRNA ligase through a two-step reaction: (1) The internal RNA 2′-phosphate reacts with NAD^+^ to form an RNA-2′-phospho-ADP-ribosyl RNA intermediate, and (2) transesterification of the ADP-ribose 2′′-OH to the 2′-phosphodiester generates 2′-OH RNA and ADP-ribose-1′′,2′′-cyclic phosphate (Spinelli et al. 1999; Steiger et al. 2001, 2005; Munir et al. 2018a). Surprisingly, TRPT1/KptA is evolutionary conserved in Archaea and Animalia, whose tRNA exon ligation does not result in a 2′-phosphate junction, as well as in bacterial species, which have no known intron-containing tRNAs and/or no known pathways to generate RNAs with internal 2′-phosphate modifications (Spinelli et al. 1998; Popow et al. 2012). These observations suggested that TRPT1/KptA might catalyze additional enzymatic reactions other than RNA 2′-phosphate removal; for example, TRPT1/KptA from *Aeropyrum pernix* and humans catalyze the NAD^+^-dependent ADP-ribosylation of either RNA or DNA 5′-monophosphate termini (Munir et al. 2018b; Munnur et al. 2019). Moreover, several PARPs are capable of ADP-ribosylating DNA or RNA ends in vitro. Among them; DNA repair PARPs (PARP1–3) can modify terminal phosphate moieties at DNA breaks with diverse specificity; i.e., PARP2 and PARP3 preferentially act on 5′-phosphates in nicked duplex DNA, whereas PARP1 modifies 3′- and 5′-phosphates as well as the terminal 2′-OH groups in single-strand or double-strand DNA (Talhaoui et al. 2016; Munnur and Ahel 2017; Belousova et al. 2018; Zarkovic et al. 2018). Beyond DNA, the antiviral PARPs 10, 11, and 15 have been shown to ADP-ribosylate phosphorylated RNA termini (Munnur et al. 2019). Although the cellular functions of this modification have so far not been investigated, it is tempting to speculate that it is involved in DNA damage repair, transcript processing, and/or defence against exogenous RNAs; e.g., of viral origin.

A group of highly diverged ARTCs, the NAD^+^:mono-ADP-D-ribosyl-DNA(guanine-N^2^)-ADP-D-ribosyltransferases, including pierisins (e.g., from *Pierisin rapae*), CARP-1 (e.g., from *Meretrix lamarckii*) and ScARP (e.g., from *Streptomyces scabies*), can directly modify guanine bases of dsDNA (Takamura-Enya et al. 2001; Nakano et al. 2006, 2013, 2015). While little is known about their physiological role, it was suggested that pierisin-1 is an important defence factor of cabbage butterflies against parasitization (Takahashi-Nakaguchi et al. 2013). Similarly, DarT, a bacterial PARP-like endotoxin, catalyzes the reversible transfer of ADP-ribose onto thymine bases of ssDNA, a process suggested to be involved in the response to adverse environmental conditions (Jankevicius et al. 2016).

### ADP-ribosylation reversal

The chemical nature of the ADPr-protein linkage as well as the length and complexity of the modification can significantly affect the PTM's half-life, the order in which downstream events occur, as well as the enzymes needed to reverse it (Alvarez-Gonzalez and Althaus 1989; Brochu et al. 1994). The hydrolysis of ADP-ribosylation linkages is carried out by members of two evolutionary distinct protein families: the macrodomains and the (ADP-ribosyl)hydrolases (ARHs). Macrodomains are both “readers” and “erasers” of ADP-ribosylation and can be evolutionary subdivided into at least six phylogenetic classes. The hydrolytically active family members are associated with either the MacroD-type (MacroD1 and MacroD2 in humans), ALC1-like (human TARG1), or PARG-like class (human PARG) (Table 1; Rack et al. 2016). Of these enzymes, MacroD1, Macro2, and TARG1 break the *O*-glycosidic ester bond of modified aspartates, glutamates, and *O*-acetyl-ADPr (*O*AADPr), the reaction product of the NAD^+^-dependent sirtuin deacetylases, as well as phosphate ester at nucleic acid ends (Sauve and Youn 2012; Rack et al. 2016; Munnur et al. 2019). PARG degrades polymers by hydrolysis of the ribose–ribose ether bond, but cannot act on the terminal protein–ribose bond (Slade et al. 2011). Three vertebrate ARH homologs were identified with ARH1 and ARH3 being confirmed hydrolases, whereas ARH2 is suspected to be catalytically inactive (Table 1; Moss et al. 1985; Oka et al. 2006; Ono et al. 2006; Smith et al. 2016; Rack et al. 2018). The available data indicate that ARH1 specifically reverses MARylation of arginine residues and appears to play a role in bacterial infections involving cholera exotoxins-like transferases (Moss et al. 1985, 1986; Kato et al. 2007). In contrast, ARH3 has a broad target spectrum including *O*AADPr, modified serine residues as well as PAR (Oka et al. 2006; Ono et al. 2006; Fontana et al. 2017; Bartlett et al. 2018). Both, ARH3 and PARG are recruited to DNA damage sites and are reported to play important parts in the DNA damage response (Mortusewicz et al. 2011; Palazzo et al. 2018; Wang et al. 2018). As for PARP1/2, this overlap in ARH3 and PARG localization and activity is yet another indication for redundancy in the ADP-ribosylation system, but may also indicate a regulatory aspect. In vitro and in vivo data suggest that PARG is the primary cellular PAR hydrolase (Alvarez-Gonzalez and Althaus 1989; Brochu et al. 1994; Fontana et al. 2017; Drown et al. 2018). However, the catalytic efficiency of PARG decreases for short polymers (less than four units) (Barkauskaite et al. 2013); hence, it is tempting to speculate whether these oligomers as well as the terminal serine linkage are the primary substrate for ARH3. This idea is supported by the fact that ARH3 knockout (KO) cells have a dramatically increased level of persistent MARylation marks, especially on histones, even in the absence of exogenous DNA damage (Fontana et al. 2017; Palazzo et al. 2018).

In addition to this complete removal of the ADP-ribosyl modification, several noncanonical mechanisms of processing have been proposed. Members of the *Legionella pneumophila* SidE effector proteins use a cascade of arginine-ADP-ribosylation on ubiquitin, phosphodiester-cleavage, and transfer of the phosphor-ribosyl-ubiquitin onto an acceptor protein as a novel ubiquitination mechanism (Bhogaraju et al. 2016; Puvar et al. 2019). Similarly, it has been demonstrated in vitro that hydrolysis of the phosphodiester bond by NUDT16, ENPP1, or snake venom phosphodiesterases leaves phosphoribosyl-modified proteins (Matsubara et al. 1970; Palazzo et al. 2015, 2016). It remains an open question whether NUDT16 and ENPP1 can process ADP-ribosylated proteins also in vivo and what the associated downstream processing or functional consequences of the phosphoribosyl modification would be.

In recent years, attention in the community has increasingly shifted toward studying erasers of ADP-ribosylation: their molecular functions, physiological roles, and influence on human health and disease. Below, we discuss these new insights into ADP-ribosylation reversing enzymes and give an overview of the structural–functional features and biological roles.

## Hydrolases of the macrodomain family

Macrodomains are evolutionarily conserved structural modules of ∼25 kDa with a typical length of 150–210 amino acids. The core motif of all macrodomains consists of a three-layer (α/β/α) sandwich architecture with a central six-stranded mixed β-sheet flanked by five α helices (Allen et al. 2003; Till and Ladurner 2009). Structurally it belongs in the leucine aminopeptidase (subunit E, domain 1) superfamily (CATH classification 3.40.220.10), which characteristically consist of nucleotide and nucleic acid-binding domains (Dawson et al. 2017). Macrodomains were shown to be binders of ADPr moieties as found in *O*AADPr, MAR-, and PARylated proteins (Karras et al. 2005). The ADPr moiety binds in a deep cleft located on the crest of the domain. Within the macrodomain family, three classes have catalytically active hydrolases as members (for review, see Rack et al. 2016).

## The PARG-like class

**Figure 2.**
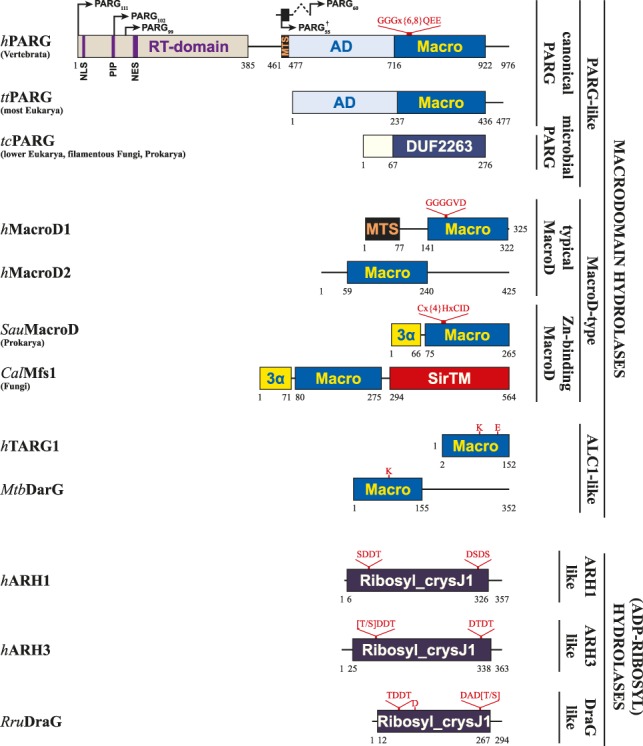
Domain structure of macrodomains and (ADP-ribosyl)hydrolases. The hydrolytic domains are Macro (macrodomain), DUF2263, (microbial PARG), and Ribosyl_crysJ1 (ADP-ribosylation/Crystallin J1 fold), respectively. Subtype-specific sequence motifs are given *above* the first domain structure (red) of its type. Canonical PARGs contain an accessory domain (AD). In vertebrata, the AD contains a mitochondrial-targeting signal (MTS) and the N terminus is extended by a regulatory and targeting domain (RT domain), which holds the nuclear localization and export signal (NLS and NES, respectively) as well as a PCNA-interacting protein (PIP) box. Other domains: 3α, 3-α-helical bundle; SirTM, sirtuin of M class. Alternative splicing of the single PARG gene in humans is indicated *above h*PARG. Note that the PARG_60_ transcript involves splicing of exons 1 and 4 as well as exclusion of exon 5 leading to an altered N-terminal sequence, but including the MTS. The arrow indicates the position from which the primary sequence corresponds to the other splice variants. (†) PARG_55_ derives from the usage of an alternative start codon in the PARG_60_ transcript.

PARGs take a special place among the macrodomains as they are the only known members to possess PAR-degrading activity (Feng and Koh 2013). In mammals, a single gene encodes alternative splice variants, which are believed to play a major role in its regulation, the subcellular distribution of de-PARylation activity as well as tissue specificity (Fig. 2; Meyer et al. 2003; Cortes et al. 2004; Meyer-Ficca et al. 2004; Cozzi et al. 2006; Niere et al. 2012). For example, PARG_111_ (isoforms are designated by the molecular weight of the corresponding protein) is a primarily nuclear protein and responsible for the degradation of PARP1/2-derived PAR following genotoxic stress (Min et al. 2010), while PARG_102_ and PARG_99_ show cytoplasmic and perinuclear localization and are thought to act on the large fraction of PAR residing in the perinuclear region (Winstall et al. 1999; Gagné et al. 2001). Furthermore, hydrolytic activity of the latter appears to be required for the regulation of PAR-induced cytoplasmic granules and protein aggregates (Grimaldi et al. 2019).

Dysfunctions in the hydrolysis of PAR chains induced by *Parg* inactivation are embryonically lethal in mice. Nevertheless, *Parg^−/−^* mouse trophoblast-derived stem cells are able to survive in the presence of chemical inhibitors of PARP1/2, suggesting that the accumulation of PAR chains, due to the absence of PARG activity, represents a cell death signal (Koh et al. 2004). Importantly, PARG depletion leads to hypersensitivity to genotoxic and replication stress and, consequently, it was proposed as a novel target for modern chemotherapeutic approaches (James et al. 2016; Palazzo and Ahel 2018; Pillay et al. 2019). In addition to its functions in DNA repair, PARG activity seems to be involved in the progression of replication forks and recovery from persistent replication stress (Illuzzi et al. 2014; Ray Chaudhuri et al. 2015). These observations are in agreement with the interaction of PARG with the replication helicase PCNA and its localization to replication foci during S-phase (Fig. 2; Mortusewicz et al. 2011; Kaufmann et al. 2017).

### Structure and function of PARG-like hydrolases

**Figure 3.**
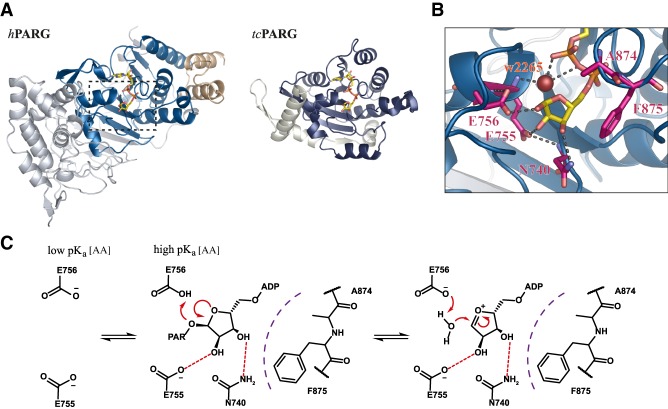
PAR degradation by PARG-like hydrolases. (*A*) Ribbon representation of the catalytic domains of canonical PARGs (depicted *h*PARG; PDB 4B1H) and microbial PARGs (depicted *tc*PARG; PDB 3SIG) in complex with ADPr. (*B*) Close up of the active site of *h*PARG. (Yellow) ADPr; (magenta) residues involved in ligand orientation and catalysis; (red) structural water (w2265); (dashed lines) selected polar interaction. (*C*) Potential reaction mechanism for PARG-like enzymes. Residue numbering is in accordance with human PARG_111_.

**Figure 4.**
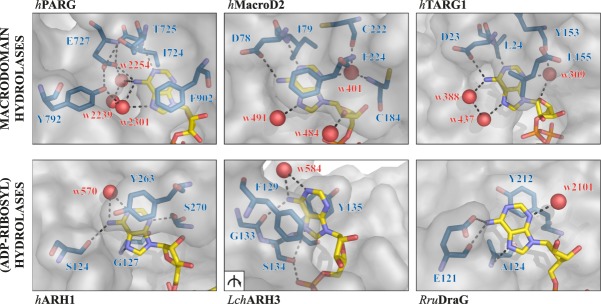
Comparison of adenine coordination across macrodomains and (ADP-ribosyl)hydrolases. Surface-liquorice representation of adenine coordination. The adenine base lies against the protein surface in most hydrolases with the exception of ARH3 in which it holds by π–π stacking perpendicular to the protein surface (view rotated [arrow] by ∼ 60° relative to the closeups). (Yellow) ADPr; (blue) coordinating residues; (red) waters; (dashed lines) selected polar contacts.

Evolutionarily, the PARG-like class can be subdivided into the canonical PARGs, found primarily in higher organisms, and the microbial PARGs, often annotated as DUF2263 (Slade et al. 2011). While the latter resemble largely classical macrodomains, canonical PARGs occur together with a mainly α-helical accessory domain that extends the core motif into a typically 10-stranded β-sheet (Figs. 2, 3A). The ADPr-binding cleft, as in other macrodomains, is part of the canonical core fold and the physiological role of the accessory domain, beyond its effects on overall protein stability, remains elusive. Within the binding cleft, the adenine moiety of the ADPr lies parallel to the protein surface and is shielded from the aqueous environment by π–π-stacking with a conserved phenylalanine (Phe902 in humans) (Fig. 4). Adenine binding is further stabilized by extensive protein and water-mediated contacts with the amino group on C6, as well as with the ring nitrogens N1 and N7 (Figs. 1, 4). These contacts convey ligand specificity as their disruption by an exchange of adenine by hypoxanthine, which substitutes the C6 amino group with a keto group, has been shown to severely diminish ADPr binding to PARG (Drown et al. 2018; Rack et al. 2018). In canonical PARGs, ligand binding is further stabilized by a highly conserved tyrosine (Tyr795 in humans) that coordinates O5′ and edge stacks with the adenosine moiety (Kim et al. 2012; Tucker et al. 2012; Lambrecht et al. 2015). Recently, these highly specific properties of the adenine-binding pocket were utilized for the development of a series of high-potency, competitive inhibitors (Waszkowycz et al. 2018). Further along the ligand, the diphosphate-binding loop coordinates both the diphosphate and distal ribose and participates in forcing a strained conformation in this part of the molecule. The strained conformation is achieved via a hydrophobic patch (G[A,V][F,Y] motif) within the loop, which bends the distal ribose toward the catalytic loop and positions C1′′ and O1′′ in relative proximity to P^α^. The conformation is further stabilized by a structural water molecule bridging the ribose and phosphate group (Fig. 1B). In canonical PARGs, a highly conserved asparagine (aspartate in microbial PARGs) precedes the catalytic GGGx{6,8}QEE motif and interacts with the 3′′OH group. Binding of the PAR substrate was suggested to increase the pK_a_ of the catalytic glutamate (Glu756 in humans), which facilitates its protonation and allows it to act as the general base in the initial step of the reaction (Fig. 1C; Slade et al. 2011; Dunstan et al. 2012; Kim et al. 2012; Tucker et al. 2012; Barkauskaite et al. 2013). The carboxyl hydrogen of Glu756 is transferred to the PAR-leaving group, while an oxocarbenium intermediate is formed on the bound distal ribose. The deprotonated Glu756 then assists in the activation of a water molecule, which reacts with the oxocarbenium intermediate and forms the ADPr product. It should be noted that due to the placement of Glu756 relative to the distal ribose, it is as yet unclear from which site the ADPr forming water attacks the ribose, and hence whether the α or β product is formed (Kim et al. 2012). Interestingly, in microbial PARGs the proximal ribose is coordinated and shielded from the aqueous environment (Slade et al. 2011), which makes this subclass strict exohydrolases. In contrast, canonical PARGs are only primarily exo-hydrolases and the more open positioning of the proximal ribose within the binding cleft allows an endo-binding mode and congruously weak endo activity has been observed in vitro (Barkauskaite et al. 2013; Lambrecht et al. 2015). However, it remains to be elucidated whether this activity is of physiological relevance.

## The MacroD-type class

Members of the MacroD-type hydrolases are widely distributed among all domains of life and several viruses (Chen et al. 2011; Rack et al. 2015, 2016; Fehr et al. 2018). In humans, the MacroD-type class has two members with highly similar catalytic domains, MacroD1 (also known as Leukaemia-Related Protein 16 [LRP16]) and MacroD2. Both enzymes are mono(ADP-ribosyl) hydrolases and active in vitro against protein substrates modified on acidic amino acids (Barkauskaite et al. 2013; Jankevicius et al. 2013; Rosenthal et al. 2013; Rack et al. 2016). Furthermore, other studies suggest that they can also hydrolyze *O*AADPr as well as (ADP-ribosyl)ated nucleic acids (Chen et al. 2011; Munnur and Ahel 2017; Agnew et al. 2018; Munnur et al. 2019). MacroD1 localizes largely to the mitochondrial matrix (Agnew et al. 2018), whereas MacroD2 distributes in the cytosol and nucleus (Jankevicius et al. 2013; Golia et al. 2017). The physiological substrates and cellular functions of both MacroD1 and MacroD2 remain largely elusive. However, links to the DNA damage response and signal transduction have been reported.

### MacroD1 and MacroD2

Aberrant *MacroD1* expression and gene fusions contribute to tumour pathology; e.g., in leukaemia, breast, gastric, liver, lung, and colorectal cancer (Imagama et al. 2007; Shao et al. 2015; Sakthianandeswaren et al. 2018). Several lines of evidence indicate that MacroD1 is involved in several important signaling pathways: In breast cancer-derived MCF-7 cells, *MacroD1* expression is induced by estrogenic hormones in an estrogen receptor alpha (ERα)-dependent manner and subsequently acts as a cofactor for ERα and the androgen receptor (Han et al. 2007; Yang et al. 2009). In response to DNA double-strand breaks, MacroD1 is activated and enriched in the cytosol, which stimulates prosurvival and antiapoptotic functions of the dimeric (p65/p50) transcription factor NF-κB (Li et al. 2017). MacroD1 stimulates the activity of NF-κB through the interaction with p65 and UXT, a transcription factor coregulator (Wu et al. 2011, 2015). In hepatocytes, MacroD1 interacts and regulates liver X receptors α and β when these are MARylated by PARP7 (Bindesbøll et al. 2016). Furthermore, MacroD1 was also proposed to act as a negative regulator of the insulin signaling pathway through the down-regulation of the insulin receptor substrate protein-1 (IRS-1) (Zang et al. 2013).

The *MacroD2* gene locus is a hot spot for mutations and chromosome rearrangements that have been associated with several human disorders, such as autism diseases, schizophrenia, and several tumors (Anney et al. 2010; Mohseni et al. 2014; Fujimoto et al. 2016; Autism Spectrum Disorders Working Group of The Psychiatric Genomics Consortium 2017). These mostly pathoneurological phenotypes of the *MacroD2* gene are associated with its loss of function, thus suggesting a physiological role in the central nervous system. This correlates with robust neuronal expression of *MacroD2* during brain development (Ito et al. 2018).

Alterations of MacroD2 functions in the DNA damage response and signal transduction may also be linked to tumor formation and/or progression. Indeed, MacroD2 is phosphorylated by ATM in response to DNA double-strand breaks, as well as being involved in reversing the ADP-ribosylation of GSK3β, a key kinase involved in the WNT-mediated signal transduction pathway (Feijs et al. 2013; Golia et al. 2017).

Despite the phenotypic and clinical associations, as well as the in vitro studies discussed above, the precise physiological roles and detailed molecular functions of both MacroD1 and MacroD2 remain poorly understood. For example, the presence of several hydrolases, including MacroD1, within the mitochondrial matrix (Fig. 2; Niere et al. 2008, 2012; Agnew et al. 2018), together with unbiased mass spectrometric evidence for ADP-ribosyl-modified proteins within this compartment (Hendriks et al. 2019) raises the question of whether ADP-ribosylation signaling has a regulatory function in mitochondria.

### Viral and microbial MacroDs

Beyond the human MacroD1 and MacroD2 proteins, viruses and bacteria encode MacroD-type hydrolases, too. MacroD-type macrodomains are encoded by a set of positive-strand RNA viruses, such as *Coronaviridae* (including severe acute respiratory syndrome [SARS-CoV] and Middle East respiratory-related coronavirus [MERS-CoV]), *Togaviridae*, and *Hepeviridae*, which all show (ADP-ribosyl)hydrolase activity against MARylated aspartate and glutamate-modified substrates (Fehr et al. 2016, 2018; Li et al. 2016; Rack et al. 2016; Eckei et al. 2017; McPherson et al. 2017; Lei et al. 2018; Leung et al. 2018; Grunewald et al. 2019). Although physiological substrates of viral MacroD-type hydrolases are not clear, they are known to be important for viral replication most likely due to their ability to counteract the host immune response by working against antiviral PARPs (PARP7, PARP9, PARP10, and PARP12–PARP15) (Atasheva et al. 2014; Li et al. 2016; McPherson et al. 2017; Fehr et al. 2018; Leung et al. 2018; Grunewald et al. 2019). This was recently corroborated by the observation that VEEV and SARS macrodomain-containing proteins can efficiently reverse PARP10-derived RNA ADP-ribosylation in vitro (Munnur et al. 2019). Noteworthy, this aspect of viral-induced stress may create evolutionary pressure and thus contribute to the rapid positive selection observed in antiviral PARPs (Daugherty et al. 2014; Gossmann and Ziegler 2014). Expression of PARP9, PARP12–14 is potently stimulated by interferon type I in response to viral infection (Juszczynski et al. 2006; Schoggins et al. 2011; Welsby et al. 2014), thus suggesting that ADP-ribosylation signaling is required for an efficient viral response. Indeed, overexpression of several PARP genes has been shown to inhibit replication of viruses (Atasheva et al. 2012, 2014). This role is partially realized through the formation of stress granules, transient cytoplasmic membraneless structures that include untranslated mRNA, specific proteins, as well as PAR, and which exhibit antiviral function among others (McInerney et al. 2005; Leung et al. 2011; Grimaldi et al. 2019). It was shown that the alphaviral macrodomain-containing nonstructural protein 3 (nsP3) interferes with the formation of stress granules and, consequently, prevents their inhibitory effect on viral replication (McInerney et al. 2005; Abraham et al. 2018). Together, these findings lead to the suggestion that targeting of viral macrodomains is a promising antiviral strategy. The hypothesis gained support recently by the development of dihydrorugosaflavonoid derivatives as inhibitors of the nsP3 macrodomain and the demonstration that these compounds are effective in reducing viral RNA levels in the infected cells (Puranik et al. 2019).

MacroD-type hydrolases are also widely spread among microorganisms, but their physiological roles have so far been understudied. However, evidence from the few studied examples suggests that these enzymes are part of the cellular stress response (Kim et al. 2008; Rack et al. 2015). For example, cold stress leads to the activation of the macrodomain YmdB in *Escherichia coli*. Subsequently, YmdB interacts with the ribonuclease RNase III and acts as a negative regulator of its cleavage activity (Kim et al. 2008; Paudyal et al. 2015). Furthermore, YmdB was suggested as a regulator of gene expression both through RNase III regulation as well as in an RNase III-independent manner, thereby influencing biofilm formation and antimicrobial resistance (Kim et al. 2013, 2017). While it was shown that YmdB is catalytically active (Chen et al. 2011; Zhang et al. 2015b), the role of this activity in vivo remains elusive. A second example of the studied microbial MacroD-type hydrolases are macrodomains associated with mono(ADP-ribosyl)transferases of the class M sirtuins (SirTMs) type that are found in bacteria (e.g., *Clostridium*, *Treponema*, and *Lactobacillus* species) and fungi (including Aspergillus, Candida, and Fusarium) (Chen et al. 2011; Rack et al. 2015). Extended operons containing a lipoyl-carrier protein (GcvH-L), a lipoyltransferase (LplA2), and the macrodomain-SirTM module are found almost exclusively in pathogenic bacteria, including *Staphylococcus aureus* and *Streptococcus pyogenes*. In this system, GcvH-L can be lipoylated by LplA2 and subsequently ADP-ribosylated by SirTM. The latter modification is reversible by the macrodomain. Interestingly, while the activity of the macrodomain is not dependent on the lipoylation, in vitro binding experiments indicate that the macrodomain interacts with GcvH-L in a lipoylation-dependent manner (Rack et al. 2015). SirTM operons in bacteria and fungi are induced by oxidative stress and it has been proposed that the lipoyl moiety acts as a reactive oxygen species (ROS) scavenger, while the ADP-ribosylation regulates its participation in the redox defence (Rack et al. 2015).

### Structure and function of MacroD-type hydrolases

**Figure 5.**
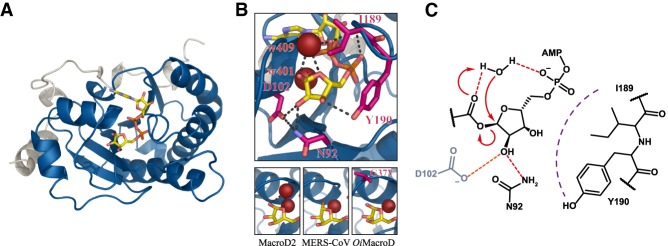
MacroD-type hydrolases. (*A*) Ribbon representation of *h*MacroD2 (PDB 4IQY) as typical representative of the MacroD-type class. (Blue) Macrodomain; (white) N-terminal extension; (yellow) ADPr. (*B*) The *top* panel shows closeup of the active site of *h*MacroD2. Color scheme as in *A*. (Magenta) Residues involved in ligand orientation and catalysis; (red) structural (w401) and catalytic water (w409); (dashed lines) selected polar interaction. The *bottom* panels show the replacement of the catalytic water from the active site in the *h*MacroD2:α-ADPr complex (PDB 4IQY), in the MERS-CoV macrodomain, due to cocrystallization with reaction product β-ADPr (PDB 5HOL), and in *Oi*MacroD, due to p.G37V mutation (PDB 5LAU). (*C*) Potential reaction mechanism for MacroD-type enzymes. Residue numbering in accordance with *h*MacroD2. Note: Asp102 is part of the proposed His/Asp dyad and is not present in all MacroD-type hydrolases.

Members of the MacroD-type class partially resemble PARG proteins with respect to their ADPr-binding features (Figs. 4, 5A,B; Barkauskaite et al. 2013). However, there are some key differences in the active site that result in very distinct catalytic mechanisms. The polymer substrate of PARG contains defined ether *O*-glycosidic bonds, whereas the linkage to acidic residues and *O*AADPr, the preferred substrates of MacroD-type enzymes, are ester linkages. One important difference is that these esters undergo spontaneous transesterification; thus, glutamyl/aspartyl of protein-linked ADPr or the acetyl moiety in *O*AADPr migrate to the 2′′ and 3′′ position and equilibrate between the three sites in a pH-dependent manner (Kasamatsu et al. 2011; Kistemaker et al. 2016). Jankevicius et al. (2013) showed experimentally and in simulations that MacroD2 cleaves ADPr from the 1′′ position (Jankevicius et al. 2013). This can be attributed to the interaction between the carbonyl group and the conserved glycine within the catalytic loop, as well as shielding of the 2′′OH-group from the environment by a conserved asparagine (Asn92 in human MacroD2) in a fashion similar to PARG (Fig. 5B). Within the catalytic loop, the consecutive, catalytic glutamate residues are absent. Initial studies of human MacroD1 and MacroD2 identified a histidine and aspartate motif on helix α6 (MacroD2) coordinating the 2′′OH, which were thought to constitute a catalytic dyad (Rosenthal et al. 2013). However, a recent study demonstrated catalytic activity of viral MacroD homologs that lack the key aspartate residue (Li et al. 2016). Taken together, these studies suggest a substrate-assisted reaction mechanism. It was proposed that P^α^ could act as a general base for activation of the structural water molecule, which would attack the C1′′ position and hydrolyze the ADPr linkage. However, this mechanism was disputed as the low pK_a_ of the phosphate group (∼2) would disfavor this reaction (Barkauskaite et al. 2013, 2015). Recent structural studies on *Oceanobacillus iheyensis* MacroD (*Oi*MacroD) identified a well-defined water molecule above the structural water that interacts with a second glycine in the catalytic loop (Figs. 2, 5B; Zapata-Pérez et al. 2017). Displacement of the water by a Gly > Val mutation reduced the catalytic efficiency of *Oi*MacroD fourfold without affecting protein stability or ADPr binding (Fig. 5B; Zapata-Pérez et al. 2017). The crystal structures of the SARS- and MERS-CoV macrodomains in complex with β-ADPr reveal occupation of the water binding site by the β-1′′OH moiety, while the structural water remains bound in the same position (Fig. 5B; Egloff et al. 2006; Lei et al. 2018), thus suggesting that this newly described water is indeed the catalytic one. Furthermore, this arrangement makes it possible to transfer the proton from the water molecule onto the leaving group or the aqueous environment. A possible, substrate-assisted S_N_2 reaction is depicted in Figure 5C, but further studies are needed to elucidate the exact nature of the transition state, mechanism, and the differences between enzymes in which the His/Asp dyad is present or absent, respectively. Comparison of MacroDs with available PARG structures revealed that the isostructural position of the catalytic MacroD glycine is not conserved but instead occupied by small aliphatic residues including alanine (*tc*PARG) or valine (*h*PARG) (Slade et al. 2011; Kim et al. 2012; Tucker et al. 2012). Consistently, no water isostructural to the proposed catalytic one can be observed in PARGs. Whether this exchange contributes to the inability of PARG to hydrolyze the terminal ADPr moiety from proteins remains, however, an open question.

Interestingly, a diverse subclass of MacroD enzymes, which are found associated with SirTMs in bacteria and fungi (Fig. 2), contain an amino acid exchange in the catalytic loop. Instead of the typical glycine-rich stretch going into helix α6 (MacroD2), these macrodomains have an extended catalytic loop containing a zinc-binding motif (Fig. 2; Appel et al. 2016). Positioning of the Zn^2+^ as part of the active site suggests a catalytic function of the ion and hence a diverged mechanism in comparison with the other members of this class.

## The ALC1-like class

Defined by a similarity to the macrodomain of the chromatin remodeler ALC1 (Ahel et al. 2009; Gottschalk et al. 2009), the ALC1-like class contains both MARylation “readers” and “erasers.” ALC1 class macrodomain proteins can be readily found in Animalia and scattered examples can be also identified amongst bacterial species (Perina et al. 2014).

### TARG1

TARG1 (also known as OARD1 and C6orf130) is the only hydrolytically active member of the ALC1-like class in Animalia. It was shown to interact with PARP1 and to possess hydrolytic activity against *O*-acyl-ADPr esters, ADPr-phosphoresters at nucleic acid termini, MARylated proteins, as well as the ability to release whole polymers from the target protein (Peterson et al. 2011; Rosenthal et al. 2013; Sharifi et al. 2013; Munnur and Ahel 2017; Munnur et al. 2019). TARG1 is found in the nucleus and cytoplasm (Sharifi et al. 2013). In particular, TARG1 has been observed to localize at the transcriptionally active nucleoli and binds strongly to ribosomes and proteins associated with rRNA processing and ribosomal assembly factors. In response to DNA damage, TARG1 relocalizes to the nucleoplasm, where it may contribute to reverse protein ADP-ribosylation (Bütepage et al. 2018).

A homozygous *TARG1* gene mutation was described in a family with 11 individuals affected by a severe and progressive neurodegeneration and seizure disorder without dysmorphic features. In detail, a premature stop codon within the exon 4 of *TARG1* locus results in the formation of a truncated and nonfunctional TARG1 protein (Sharifi et al. 2013). In addition, a genome-wide association study revealed that the *TARG1* gene could be associated with the loss of insulin sensitivity, a key factor contributing to metabolic disease. However, a functional link between TARG1 and the cellular insulin response has at yet not been established (Timmons et al. 2018).

### DarG

DarG is a member of the ALC1-like macrodomains found strictly as a two-component toxin–antitoxin operon in a variety of bacteria, including pathogens like *Mycobacterium tuberculosis*, enteropathogenic *E. coli*, and *Pseudomonas aeruginosa*, as well as several hyperthermophiles such as *Thermus aquaticus* (Sberro et al. 2013; Jankevicius et al. 2016). The toxin DarT, a Bc4486-like member of the PARP family (de Souza and Aravind 2012; Aravind et al. 2015), modifies ssDNA at thymine bases in a sequence-specific manner (Jankevicius et al. 2016). The formation of the (ADP-ribosyl)-DNA adduct is reversed via the action of the antitoxin DarG, which shares some functional features with TARG1 (Jankevicius et al. 2016). As such, DarTG represents the first characterized system for the reversible ADP-ribosylation of nucleic acids. While the exact physiological role of DarTG is unclear, it was shown that the toxin blocks DNA replication, and it has been speculated that the host bacteria may exploit this system in order to induce a persistence state to survive adverse environmental conditions including exposure to antibiotics (Jankevicius et al. 2016). If true, resuming growth would require DarG antitoxin activity, which would be in line with *M. tuberculosis* transposon mutagenesis studies indicating that DarG is an essential gene (Sassetti et al. 2003; Griffin et al. 2011). Taken together, the inhibition of DarG may present a new and promising therapeutic strategy to combat bacterial infections (Jankevicius et al. 2016).

### Structure and function of ALC1-like hydrolases

**Figure 6.**
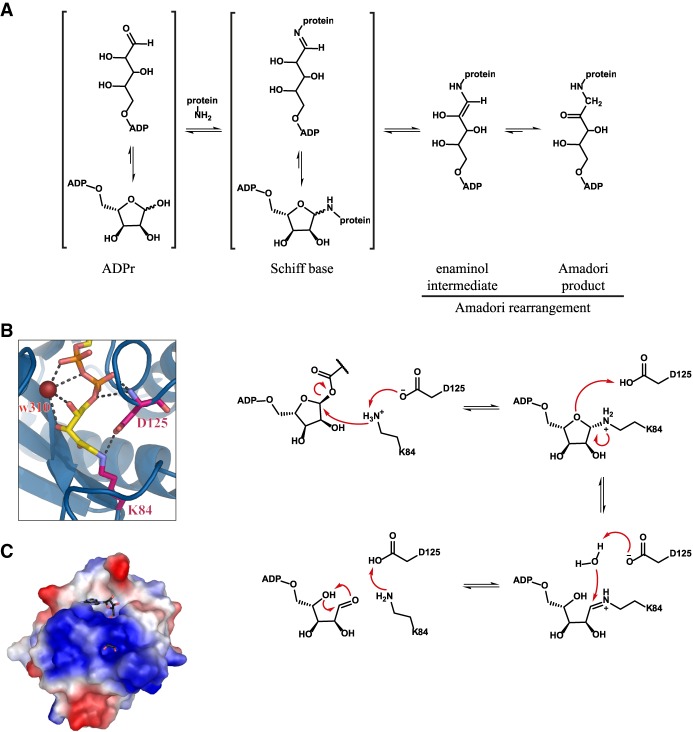
ALC1-like hydrolases. (*A*) Reaction mechanism for the nonenzymatic formation of a Schiff base and the Amadori rearrangement. (*B*) Closeup of the active site of *h*TARG1 in complex with the Amadori product of ADPr (yellow) and Lys84. (Magenta) Catalytic residues; (red) structural water (w310); (dashed lines) selected polar interaction. (*C*) Proposed reaction mechanism for TARG1 and related ALC1-like hydrolases. Residue numbering in accordance with *h*TARG1. (*D*) Electrostatic surface map of *T. aquaticus* DarG. (Red) Negative surface charge; (blue) positive surface charge; (white) neutral surface charge. Note that the prominent positively charged area, which runs perpendicular to the active site, was suggested as the DNA-binding surface. The cocrystallized ADPr is depicted in CPK coloring.

In their overall structure, ALC1-like macrodomains are minimal without C- or N-terminal extensions and only five α-helices (Fig. 2). The most considerable divergence to other macrodomain hydrolases is, however, their catalytic mechanism. Crystal structures of the TARG1:ADPr complex showed that in crystallo Lys84 of TARG1 reacts with the distal ribose C1′′ forming an open ring Amadori product (Fig. 6A,B; Sharifi et al. 2013). Further functional analysis of this residue revealed that it is together with Glu125 part of a catalytic dyad. Interestingly, mutation of Glu125 leads in vitro to the formation of a covalent reaction intermediate, indicating that the hydrolytic mechanism also proceeds through a covalent intermediate, which is resolved by Glu125 (Sharifi et al. 2013). Therefore, the authors suggested a reaction mechanism resembling that of 8-oxoguanine DNA glycosylase (OGG1) (Bruner et al. 2000). Such a mechanism would involve deprotonation of Lys84 by Glu125 and an attack of the nitrogen onto the anomeric carbon with liberation of the modified glutamate/aspartate (Fig. 6B,C). Subsequently, the ribose of the resulting *N*-gloycosidic intermediate opens up to form a Schiff base, which is susceptible to a nucleophilic water attack. This step leads to the formation of a ring-opened ADPr and enables regeneration of the catalytic lysine. While the mechanism explains neatly the observations and fits well with similar mechanism in other systems, two problems remain: First, the positioning of ADP-HPD and ADPr in the available structures resembles the binding mode of ADPr in MacroD-type and PARG-like macrodomains, and in this binding position the anomeric carbon is not available for the initial attack by the catalytic lysine residue. Second, the spontaneous transesterification of the substrate makes it possible that the hydrolysis could occur from the 2′′ or 3′′ position, and so far no experimental evidence is available to determine from which position the modification is cleaved. In order to reconcile the positioning of the ribose within the active site, it was suggested that binding of the substrate is directed by the TARG1 diphosphate-binding loop, which would result in an alternative ribose conformation permissive for cleavage (Sharifi et al. 2013). Support for this comes from the DarG antitoxin structure in which a positive surface patch, presumably the ssDNA-binding site, runs perpendicular to the ADPr binding pocket (Fig. 6D; Jankevicius et al. 2016). This allows for the speculation that the distal ribose is orientated toward the catalytic residue. Whether such a reorientation occurs for small substrates such as *O*AADPr and whether hydrolysis indeed occurs from the C1′′ position remains, however, a subject for future studies. It is also of note that DarG does not contain the catalytic Lys/Glu dyad and only the lysine residue remains conserved between the two enzymes (Jankevicius et al. 2016). Absence of both residues was noted in SCO6735, an ALC1-like hydrolase from *Streptomyces coelicolor* involved in antibiotic production (Lalić et al. 2016). This further indicates a major mechanistic diversification within the ALC1-like class. Taken together, important insights into this class of hydrolases have been achieved in recent years, but important questions remain: What are the mechanistic similarities and differences between TARG1, DarG, and SCO6735? Is this diversification within the ALC1-like class associated with a physiological function or necessity?

## The (ADP-ribosyl)hydrolase family

The ARH family is an evolutionary highly conserved structural module adopting a mainly α-orthogonal bundle architecture with a typical domain length of 290–360 residues. The first family member was identified as an activating factor, now termed DraG, which reverses the arginine-ADP-ribosylation-inhibiting dinitrogenase reductase (Fe-protein) in *Rhodospirillium rubum* (Ludden and Burris 1976). An enzyme with the same activity and comparable properties, now known as ARH1, was later identified in animal cells (Moss et al. 1985).

### ARH1

ARH1 is a cytoplasmic protein, which is ubiquitously expressed in human and mouse tissues (Moss et al. 1992). Its primary activity is the hydrolysis of the *N*-glycosidic arginine-ADPr bond and has negligible activity against PAR and *O*AADPR (Oka et al. 2006; Ono et al. 2006; Mashimo et al. 2014; Rack et al. 2018). Deficiency of *Arh1* in mouse embryonic fibroblasts (MEFs) and tissues dramatically impairs the ability to hydrolyse endogenously produced arginine-modified substrates (Kato et al. 2007), suggesting that ARH1 is the main cytoplasmic enzyme carrying out this reaction. Although the physiological role of ARH1 is not well understood, phenotypic observation on *Arh1^−/−^* mice and derived *Arh1*-deficient MEFs suggest a leading role of ARH1 in intracellular signal transduction and cell cycle regulation. Indeed, depletion of *Arh1* in MEFs led to an abnormal proliferation rate characterized by a shortened G1 phase and rapid cell growth compared with wild-type MEFs (Kato et al. 2011). Consequently, it was observed that *Arh1*^−/−^ and *Arh1*^+/−^ mice have an increased risk of developing several types of tumors, including carcinoma, sarcoma, and lymphoma (Kato et al. 2011). Notably, estrogens play a key role in tumourigenesis observed in *Arh1*^−/−^ mice and MEFs, thus showing a significant gender-specific phenotype (Shim et al. 2013). The involvement of ARH1 in cancer progression is confirmed by the observation of frequent human somatic mutations in the *ARH1* gene in lung, breast, and colon cancers (Kato et al. 2015). Some of these mutations directly impact the catalytic activity; e.g., the p.D56N missense mutation affects Mg^2+^ coordination and inactivates ARH1 (Kato et al. 2015; Rack et al. 2018).

In addition, ARH1 plays a role in the protection from *Vibrio cholera* infections (Kato et al. 2007; Watanabe et al. 2018). Cholera toxin, which is secreted during infection, inhibits the GTPase activity of the α subunit of the stimulatory guanine nucleotide-binding (G_S_α) protein by MARylation of an arginine residue, thus maintaining G_S_α’s active form. This results in accumulation of intracellular cAMP, ultimately leading to abnormalities in fluid and electrolyte transport that are the hallmark of *Vibrio cholera* pathogenesis (Vanden Broeck et al. 2007; Catara et al. 2019). *Arh1^−/−^* mice exhibit enhanced sensitivity to the toxin with significantly increased fluid accumulation in the intestinal loops (Kato et al. 2007; Watanabe et al. 2018). Moreover, a crosstalk between arginine- and serine-ADP-ribosylation has been recently reported. Specifically, exposure of cultured cells to cholera toxin caused formation of free arginine-ADPr (Arg-ADPr), as also demonstrated earlier in vitro (Oppenheimer 1978), which then specifically inhibits the ARH1 homolog ARH3 with nanomolar affinity (Drown et al. 2018; Rack et al. 2018). ARH1 can degrade free Arg-ADPr in vitro (Moss et al. 1986), and congruously, withdrawal of the exotoxin from the culture media restores ARH3 activity (Drown et al. 2018). Whether inhibition of ARH3 during infection involving cholera toxin-like enzymes is part of the bacterial virulence (e.g., by altering the cellular DNA damage response) remains, however, to be clarified.

### ARH3

ARH3 is a ubiquitous protein conserved in Animalia and Capsaspora (Oka et al. 2006). ARH3 localizes to the cytosol, mitochondria, and nucleus, and experimental data suggest that the precise subcellular distribution may depend on cell type as well as cellular requirement (Oka et al. 2006; Niere et al. 2012; Mashimo et al. 2013). For example, ARH3 was detected in the nuclei of mouse brain and MEFs, but was absent in the ones of HepG2 cells (Oka et al. 2006; Mashimo et al. 2013; Bonfiglio et al. 2017), which suggests that ARH3 may have cell type-specific functions.

ARH3 has a key role in the hydrolysis of serine-linked ADPr that is used in regulation of numerous proteins controlling genome stability in higher organisms (Abplanalp et al. 2017; Bonfiglio et al. 2017; Fontana et al. 2017; Palazzo et al. 2018). In vitro studies with all known (ADP-ribosyl)hydrolases indicate that for this function no backup pathway exists in mammalian cells (Fontana et al. 2017). In addition, hydrolysis of PAR chains as well as *O*AADPR has been reported for ARH3 (Oka et al. 2006; Kasamatsu et al. 2011; Mashimo and Moss 2016; Fontana et al. 2017), but in this case, alternative hydrolases exist in the cells. PAR-removing activity of ARH3 has been linked to the regulation of parthanatos, a special type of apoptosis (Mashimo et al. 2013; Dawson et al. 2017; Robinson et al. 2019).

The partial redundancy between PARG and ARH3 and the preference for serine-linkages, the most prevalently modified residue in the DNA damage response, suggests a prominent role for those enzymes in the maintenance of genome stability (Mashimo et al. 2013, 2019; Tanuma et al. 2016; Fontana et al. 2017; Palazzo et al. 2018). The increased sensitivity of human and mouse ARH3-deficient cells to hydrogen peroxide-induced cell death supports this theory (Tanuma et al. 2016; Palazzo et al. 2018). Loss-of-function mutations in *ARH3* were linked to the pathogenesis of a rare recessive autosomal neurodegenerative disorder (Danhauser et al. 2018; Ghosh et al. 2018), suggesting that ARH3 contributes to the protection of neurons from endogenous ROS. In contrast, the mitochondrial function of ARH3 remains elusive, but current observations support two possibilities: First, ARH3 can degrade ADP-ribosylation artificially targeted to the mitochondrial matrix, and hence may be responsible for potential endogenous ADP-ribosylation in this compartment (Niere et al. 2012). Second, the ability to degrade *O*AADPr suggests a role of ARH3 in metabolite salvage and NAD recycling (Dölle et al. 2013).

### DraG

Several bacteria as well as a few archaea, collectively termed diazotrophs, have the ability to convert atmospheric, molecular nitrogen into ammonia, thus making it available for the biosphere. Due to the high energetic costs associated with this process, its tight regulation is crucial. Some diazotrophs control the pivotal nitrogenase complex by reversible ADP-ribosylation of the Fe-protein, also known as the dinitrogenase reductase component. Through dedicated investigation over the last decades, this system has become one of the best-studied reversible ADP-ribosylation signaling pathways. The Fe-protein homodimer is ADP-ribosylated at a single arginine residue (Arg101 in *Rhodospirillum rubrum* DraG [*Rru*DraG]) by the ARTC family member DraT (Pope et al. 1985; Ma and Ludden 2001). This prevents formation of the nitrogenase complex, which consequently reduces nitrogen fixation. The modification is reversed by (ADP-ribosyl-[dinitrogenase reductase])hydrolase DraG (Ludden and Burris 1976; Saari et al. 1984). Furthermore, the system is controlled by members of the P_II_ nitrogen regulatory protein family, which directly and indirectly sense a variety of negative stimuli, including high ammonia or glutamine, low cellular energy, or absence of light (Huergo et al. 2012; Nordlund and Högbom 2013). The cellular energy status is “read” by the P_II_ proteins GlnB and GlnK (orthologous also called GlnZ), which competitively bind ATP and ADP in a cleft at the homotrimer interphase (Xu et al. 1998; Jiang and Ninfa 2007). In vitro studies have shown that in the ADP-bound state GlnB associates with DraT, which results in its activation. Concurrently, the P_II_ protein GlnK:ADP complex associates with DraG, leading to its partial inhibition, and further full inactivation is achieved by association of this ternary complex with the ammonia transporter AtmB, hence sequestering DraG at the cellular membrane (Rajendran et al. 2011; Moure et al. 2019). Jointly, these processes lead to inactivation of the nitrogenase complex. Binding of ATP to GlnB and GlnK is synergistic with 2-oxogluterate, a cellular signal of nitrogen and carbon status, (Jiang and Ninfa 2007) and leads to dissociation of DraT and DraG and activation of the nitrogen fixation pathway (Gerhardt et al. 2012; Nordlund and Högbom 2013). It is noteworthy that this represents only one aspect of nitrogen fixation regulation and the system can be further fine-tuned by uridylylation of the P_II_ proteins as well as transcriptional regulation of components of the nitrogen fixation pathway (Huergo et al. 2012; Nordlund and Högbom 2013).

### Structure and function of ARH enzymes

**Figure 7.**
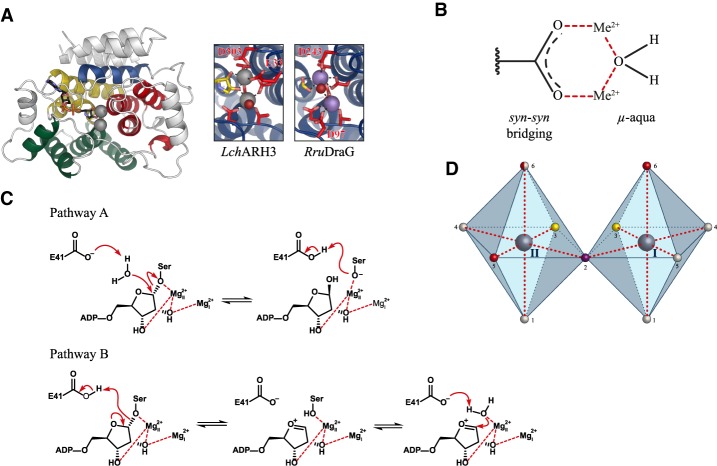
ARH structure and mechanism. (*A*) The *left* panel shows a ribbon representation of *Lch*ARH3 in complex with the ADPr analog ADP-HPD (CPK coloring; PDB 6HH3). The conserved 13 α-helical core motif is colored according to quasidomain classification. (Red) A; (green) B; (yellow) C; (blue) D. The *right* panels show a closeup of the metal coordination of *Lch*ARH3 in complex with Mg^2+^ (dark gray) and *Rru*DraG in complex with Mn^2+^ (mauve). (*B*) Schematic representation of metal coordination defining the metal-to-metal distance. (C) Schematic representation of the dinuclear metal center. Both metals (dark gray) are octahedral coordinated. Ligands in the first coordination sphere are protein-derived monodentates (white), water (red), *μ*-aqua (purple), and *syn*–*syn*-bridging carboxyl (yellow). Note that axial position 6 of Me_II_ can be occupied by either water or glutamate, depending on the conformation of the Glu flap. (*D*) Potential reaction mechanisms for ARH3-type enzymes. Residue numbers according to *h*ARH3.

Structurally, ARH proteins are compact and globular with a central core motif consisting of 13 orthogonal α-helices and a variable number of auxiliary helices depending on the organism and type (e.g., total number of helices: 25 in *h*ARH1 [PDB 6G28], 22 in *h*ARH3 [PDB 2FOZ], and 18 in *Rru*DraG [2WOD]). The overall fold can be subdivided into four quasidomains with the ADP-ribose binding site as well as the catalytic binuclear metal center embedded into their interphase (Fig. 7A; Mueller-Dieckmann et al. 2006; Li et al. 2009; Rack et al. 2018). Coordination of the adenosine moiety differs greatly between the different ARH classes. In DraG, the adenine moiety is coordinated parallel to the protein surface and stacks on top of a conserved tyrosine residue (Tyr212 in *Rru*DraG). Exact positioning is achieved by interaction of the C6 amine and N7 nitrogen with a conserved ExxA motif (Glu121 in *Rru*DraG). The proximal ribose makes no contacts within the binding cleft and the 2′ and 3′ OH groups are orientated toward the aqueous environment. In ARH1, the human functional equivalent of DraG, the adenosine is likewise parallel to the protein surface; however, it is shielded from the environment by π–π stacking with a conserved tyrosine residue (Tyr263 in *h*ARH1). While comparable coordination of the C6 amine N7 nitrogen can be observed in the *h*ARH1 structure, the corresponding residues (Ser124 and Gly127 in *h*ARH1) are not well conserved among ARH1's (Rack et al. 2018). The 2′ and 3′ OH groups of the proximal ribose interact with an ARH1-specific loop region, termed the adenosine-binding loop (Rack et al. 2018). In ARH3, the adenine moiety is orientated perpendicular to the protein surface and stacked between two conserved aromatic residues (Phe143 and Tyr149 in *h*ARH3). As in DraG, the hydroxyl groups of the proximal ribose are exposed to the environment. This orientation is compatible with both endo- and exo-PAR hydrolysis, yet ARH3 endo activity has not been demonstrated so far.

All ARH-type enzymes characterized so far are activated by divalent metal ions coordinated within a binuclear metal center (Nordlund and Norén 1984; Moss et al. 1985; Antharavally et al. 1998; Oka et al. 2006). The residues involved in metal coordination are highly similar, but subclass-specific motifs could be identified (Fig. 2; Mueller-Dieckmann et al. 2006; Berthold et al. 2009; Li et al. 2009; Rack et al. 2018). Dependence on the nature of the divalent-cation was investigated for DraG and ARH3: DraG primarily uses Mn^2+^, with its activity also supported by Fe^2+^ and, to a lesser extent, Co^2+^ and Mg^2+^ (Nordlund and Norén 1984; Ljungström et al. 1989). In contrast, ARH3 primarily uses Mg^2+^, but can also be activated by Mn^2+^ (Rack et al. 2018). No detailed investigation for ARH1 was so far carried out, but it is known that Mg^2+^ will support its activity (Moss et al. 1985). In the unligated state, the coordination spheres of the two divalent ions are connected by a *syn*–*syn* bridging aspartate (Asp316 *h*ARH3) as well as a *μ*-aqua ligand (Fig. 7B,C). The latter is displaced upon substrate binding by the 2′′OH group of the distal ribose both in *h*ARH1 and *Lch*ARH3 (Rack et al. 2018). In crystallo, *h*ARH3 can coordinate ADPr even in presence of the *μ*-aqua ligand albeit with unusually short coordination bonds (Pourfarjam et al. 2018; Wang et al. 2018). Therefore, details of the ligand binding under more physiological conditions remain to be elucidated. However, it is clear that the correct positioning of the substrate in the active site requires both metal ions to be present as well as the *cis* 2′′ and 3′′ OH groups of the distal ribose of the substrate (Pourfarjam et al. 2018; Rack et al. 2018; Wang et al. 2018). The observed arrangement of ligands in the active site also gives a structural explanation for the observed selectivity toward α-1′′-linkages by ARH1 and ARH3 (Moss et al. 1986; Voorneveld et al. 2018). Interestingly, ligand binding was also associated with conformational changes near the active site: One of the axial positions of the metal ion II (Me_II_) shows flexible occupation either by an *μ*-aqua ligand or glutamic acid residue (Glu41 in *h*ARH3) (Fig. 7C). The loop containing the latter, termed the Glu flap, can undergo conformational changes and it was proposed that coordination of Mg^2+^ in ARH3 by Glu41 represents as a closed, self-inhibitory state, and that displacement of the loop is a prerequisite for substrate binding (Pourfarjam et al. 2018). In addition to the conformational change, the glutamate residue is crucial for enzymatic activity of ARH3 (Mueller-Dieckmann et al. 2006; Abplanalp et al. 2017; Rack et al. 2018). Beyond this common set of metal coordination features, DraG enzymes contain an additional, highly conserved aspartate (Asp97 in *Rru*DraG; absent in ARH1 and ARH3) in proximity of Me_I_. While direct contacts with a cocrystallized Mn^2+^ ion could be observed in *Rru*DraG (Berthold et al. 2009), the interaction was absent in a structure of the *Azospirillum brasilense* homolog (*Abr*DraG) in complex with Mg^2+^ (Li et al. 2009).

So far only one structure of a DraG-type hydrolase in complex with ADPr is available (Berthold et al. 2009). The electron density of the *Rru*DraG:ADPr complex showed an Amadori product similar to TARG1 (Fig. 6A). However, in contrast to TARG1, the lysine reacting with the active site-bound ADPr is donated from a neighbouring protomer in the crystal packing rather than part of the active site itself. Secondly, it was shown that mutating Glu28 (*Rru*DraG), the structural homolog residue of the Glu flap glutamate, has only a minor effect on catalytic activity, while Asp97 is crucial (Berthold et al. 2009). Comparison of the structures of *Rru*DraG and *Abr*DraG reveals stark differences in terms of metal coordination: While *Abr*DraG adopts a coordination similar to ARH1 and ARH3 (see above; Li et al. 2009), in the *Rru*DraG structure the geometry appears to be rotated by ∼90°, which results in an axial positioning of the *μ*-aqua ligand (Fig. 7A,C). In this conformation, the *μ*-aqua can act as a nucleophile attacking the Schiff base intermediate at C1′′ (Berthold et al. 2009). However, the geometry observed in the *Rru*DraG crystal structure does not include a bridging carboxyl group as predicted from earlier electron spin resonance measurements and observed in all other ARH structures (Antharavally et al. 1998; Mueller-Dieckmann et al. 2006; Li et al. 2009; Pourfarjam et al. 2018; Rack et al. 2018; Wang et al. 2018). While details of the DraG mechanism remain elusive, the data point to a prominent role of Me_I_ in the reaction mechanism.

ARH1, the functional homolog of DraG, is mechanistically even less understood, but first structural insights have been gained recently (Rack et al. 2018). Its structural features are a hybrid of DraG and ARH3 with the absence of the DraG-specific aspartate (Asp97 in *Rru*DraG; similar to ARH3) as well as increased constrains on the Glu flap flexibility (similar to DraG) (Pourfarjam et al. 2018; Rack et al. 2018). Further studies are needed to understand the hydrolytic mechanism and reveal how far the similarities between ARH1 and the other ARH classes stretch.

The recently solved structures of ARH3 lead to the proposal of different catalytic mechanisms (Pourfarjam et al. 2018; Rack et al. 2018; Wang et al. 2018). Absence of the *μ*-aqua in ligand-substituted ARH3 structures indicates that it is dispensable for the catalytic mechanism (Rack et al. 2018). However, the available data point toward a closer engagement of the substrate with Mg_II_ and a more structural role for Mg_I_ (Pourfarjam et al. 2018; Rack et al. 2018). Furthermore, computational modeling and biochemical evidence suggest that the axial water in position 6 (Fig. 7C) is displaced with the C1′′ substituent, which is either the *O*-glycosidic serine linkage or 1′′ scissile bond of PAR. In this conformation, the β face of the distal ribose would be accessible for a nucleophilic attack of a Glu flap-activated water molecule. This would lead to an S_N_2-type reaction intermediate and formation of an oxyanion (Fig. 7D). Alternatively, direct protonation of the leaving group by Glu41 is possible and would result in the formation of an oxocarbenium intermediate (Fig. 7D). Further studies focusing on the interaction with true substrates are needed to elucidate the details of the reaction mechanism.

## Reversal of nucleic acid ADP-ribosylation

Within the realm of ADP-ribosylation signaling, modification of DNA and RNA phosphor-termini is a newly emerging field of study (Talhaoui et al. 2016; Munnur and Ahel 2017; Munir et al. 2018b; Munnur et al. 2019). While the cellular functions are as yet elusive, the association of this modification with DNA repair as well as antiviral PARPs suggests functions in DNA damage repair and antiviral defence. One possibility is that DNA ADP-ribosylation may act as a reaction intermediate similar to DNA adenylation during DNA ligation (Lehnman 1974; Pascal 2008; Tanabe et al. 2015). This hypothesis is particularly interesting, as a recent study suggests that human DNA ligase IV, involved in damage repair, can use NAD^+^ (Chen and Yu 2019). Alternatively, capping of 5′ phosphates could have a protective function to preserve the phosphorylation until the required repair factors are assembled at the damage site. In contrast, presence of a 3′-phosphate can interfere with efficient repair and it has been suggested that *E. coli* primes such position for repair by attachment of a guanyl-cap (Chauleau et al. 2015). As for RNA, ADP-ribosylation may contribute to the recognition and/or processing of exogenous and hazardous RNAs; e.g., transposon-derived noncoding or viral RNAs.

Regardless of the exact physiological role, the modification of 3′- and 5′-phosphor termini is reversible by the action of PARG, MacroD1/2, TARG1, and ARH3 (Munnur et al. 2019). This diversity of enzymes capable of removal may be surprising given the diversity of hydrolytic mechanisms discussed above. However, this may at least partially be the result of the inherent properties of the enzymes and the substrate: (1) a high degree of accessibility of DNA/RNA ends relative to most modifications confined within a protein structure; (2) formation of the phosphate product is favorable in comparison with other reaction intermediates, thus supporting hydrolysis; and (3) ARH3 as well as macrodomains bind ADPr with high affinity and hence are predicted to interact with ADPr adducts readily as long as the linked group does not clash with the structure of the hydrolase. Together, the relative nonspecificity of ADPr hydrolysis from nucleic acid termini suggests that it is regulated through recruitment or exclusion of hydrolases from the cellular context in which this modification occurs, but further studies are needed to elucidate the exact similarities and differences in the hydrolysis catalyzed by the various enzymes as well as the exact nature of their regulation.

## Conclusions and perspectives

The examples discussed in this review reflect the increasingly compelling view that (ADP-ribosyl)hydrolases deserve a more prominent role in the investigation of ADP-ribosyl signaling. Understanding their molecular function and substrate specificities will allow us to link them more conclusively to the specific ARTs and thus create a direct functional relationship between “readers” and “writers.” Beyond the immediate biochemical connection, it is our hope that future studies will use these links to elucidate the role of the hydrolases in their specific signaling pathways. In this context, it is important to note that the study of hydrolases should be extended beyond the human realm since many (ADP-ribosyl)hydrolases in plants, pathogenic organisms, and model systems among others have still unclear functions (de Souza and Aravind 2012; Perina et al. 2014; Aravind et al. 2015; Zhang et al. 2015a; Gunn et al. 2016; Haikarainen and Lehtiö 2016; Lalić et al. 2016; Zapata-Pérez et al. 2017).

Future efforts in the development of small molecule inhibitors will hopefully produce new probes to study the (patho-)physiological roles of these fascinating enzymes as well as lead to new drugs with therapeutic applications. The potential of such an approach was highlighted over recent years with the development of PARG inhibitors finding their application in cancer therapy (James et al. 2016; Gravells et al. 2017; Waszkowycz et al. 2018).
